# Effects of Mating on Gene Expression in Female Insects: Unifying the Field

**DOI:** 10.3390/insects13010069

**Published:** 2022-01-07

**Authors:** Ferdinand Nanfack-Minkeu, Laura King Sirot

**Affiliations:** Department of Biology, The College of Wooster, Wooster, OH 44691, USA; fnanfackminkeu@wooster.edu

**Keywords:** mating, gene expression, chemosensation, metabolism, immune response

## Abstract

**Simple Summary:**

Insects play many important roles including in ecosystems, food production, pathogen transmission, and production of materials. As a result, humans are interested in understanding how to control insect population sizes for control, propagation, or conservation efforts. In many insect species, female reproductive output is promoted by mating and components of the ejaculate. Beyond just the impact of receiving sperm, mating and ejaculate components can result in increased rate of oocyte development, ovulation, and oviposition as well as other changes such as reduced mating receptivity. To understand how mating causes these changes, researchers have investigated changes in female gene expression that occur after mating. In this review, we summarize the current state of knowledge on mating-induced gene expression changes in female insects and the methods used for conducting such studies. We find that genes related to immune response, chemosensation, and metabolism are commonly regulated across species. We suggest future research paths to facilitate the comparison of studies on mating-regulated gene expression across insect species.

**Abstract:**

There is intense interest in controlling insect reproductive output. In many insect species, reproductive output is profoundly influenced by mating, including the receipt of sperm and seminal fluid molecules, through physiological and behavior changes. To understand these changes, many researchers have investigated post-mating gene expression regulation. In this review, we synthesize information from studies both across and within different species about the impact of mating, or components of mating, on female gene expression patterns. We found that genes related to the roles of metabolism, immune-response, and chemosensation are regulated by mating across many different insect species. We highlight the few studies that have taken the important next step of examining the functional consequences of gene expression regulation which is crucial in order to understand the mechanisms underlying the mating-regulated control of female lifespan and reproduction and to make use of such knowledge to propagate or control insect populations. The potential of cross-study comparisons is diminished by different studies using different methods. Thus, we also include a consideration of how future studies could be designed to facilitate cross-study comparisons and a call for collaboration across researchers studying different insect species and different aspects of insect biology.

## 1. Introduction

Insects play important roles in human society. Some of these roles are beneficial such as providing pollination and sources of silk and food; others are detrimental such as acting as pests of crops and vectors of disease-causing pathogens [1,2,3]. For both of these categories, as well as other insect species of conservation concern, humans may want to manage a species’ reproductive output for a variety of reasons including to increase agricultural productivity, decrease pathogen spread, and/or increase endangered or threatened populations.

New advances for managing insects could come from understanding how mating impacts key behavioral and physiological processes. Across different insect species, mating modifies several female phenotypes including feeding, digestion, egg production, immune response, locomotion, olfactory and visual responses, mating receptivity, morphology, and lifespan (e.g., [4,5,6,7,8,9,10]. Similar phenotypic changes can occur across insects with different mating systems. For example, both in species in which females mate only once on average during their lifetime (e.g., *Aedes aegypti* and *Anopheles gambiae;* Diptera: Culicidae)) and species in which females mate multiple times (e.g., *Drosophila melanogaster*; Diptera: Drosophilidae), mating results in reduced receptivity to future inseminations [11]. The cues that induce post-mating changes in females could include internal and external physical stimulation and internal and external biomolecules (e.g., pheromones and seminal fluid molecules). For some species, particular cues that females receive during mating have been identified as contributors to these phenotypic changes (e.g., sex peptide in *D. melanogaster*) [6,12]. These cues include the receipt of sperm and/or seminal fluid molecules (SFMs; e.g., [4,7,13], which can be transferred in the form of liquid ejaculate or more solid spermatophores and mating plugs [14]. However, the mechanisms by which females respond to these cues to produce phenotypic changes are still largely unknown. For example, although 292 seminal fluid proteins (SFPs) have been identified in *D. melanogaster* [15], the receptor in the female for only one of them (sex peptide) has been definitively identified [16,17,18]. The detailed mechanisms of how mating-induced phenotypic changes occur has rarely been established [19,20].

One approach to begin to understand the mechanisms underlying female mating-induced phenotypic changes is to study mating-induced gene expression changes. Such studies have been conducted in several insect species and have revealed interesting patterns. Some expression changes are in genes with predicted functions related to known female post-mating changes (e.g., egg production, immunity, and chemosensation; [21,22,23,24,25], whereas others are in genes with unexpected predicted functions (e.g., muscle development, pH regulation; and translation initiation factors; [26,27,28] which have led to new candidate phenotypes to test for post-mating changes. However, most such studies have resulted in as-of-yet untested hypotheses about the connections between mating-regulated genes and mating-regulated phenotypes. As a result, the full potential for such studies to result in a deeper understanding of basic biology, to discover novel mating-regulated phenotypes, and to generate novel applications for insect management has yet to be realized. Furthermore, due to variation in the methodologies used in the different studies, it has been difficult to draw any conclusions about commonalities and differences between species in how mating or components of mating (e.g., receipt of sperm or SFMs) impact female gene expression. In this paper, we synthesize the current state of knowledge about genes and pathways regulated by mating or components of mating in female insects in order to identify apparent commonalities across species. Most of the published studies on this topic are from just a few species of Diptera and Hymenoptera, although we include all species for which we found studies. Further, we review the different methodologies used to address the question of how mating impacts female gene expression and provide a framework for consistent methodology that will allow for more meaningful comparisons across studies in the future.

## 2. Commonalities in Mating-Induced Gene Expression Changes across Species

Across different studies of mating-regulation of female gene expression (sometimes using different methods), we see some commonalities emerge in the predicted or known functions of regulated genes, as well as apparent species- (or study-) specific differences. Metabolism, immunity, and chemosensation are the main functions (or predicted functions) of the proteins encoded by genes regulated by mating across multiple insect species. These findings correspond to findings from studies of SFMs which have revealed that males transfer proteins to females known or predicted to influence these same processes [4]. Therefore, we will review findings on genes in these three functional groups before discussing two species-specific cases of studies that have taken the next important step of connecting the mating-regulated gene expression changes with post-mating physiological and behavioral changes in females.

**A.**  **Metabolism-related genes**

Metabolic processes are mainly used by insects to produce energy and to obtain necessary metabolites that can be important for many processes including development, immune response, reproduction, and locomotion. Gene ontology analysis have shown an enrichment of metabolism-related genes of those genes regulated by mating in female *Apis mellifera* (Hymenoptera: Apidae), *Anastatus disparis* (Hymeonptera: Eupelimidae), *Ae. aegypti*, *Bactrocera dorsalis* (Diptera: Tephritidae), *Callosobruchus maculatus* (Coleoptera: Chrysomelidae), *D. melanogaster*, *An. gambiae* [25,29,30,31,32,33,34,35,36,37]. The main metabolism-related families regulated by mating in females across different insect species are oxidoreductases, hydrolases and transferases [26,29,31,34,37,38]. Although the function of the regulation of these genes have not been investigated, based on post-mating behavioral and physiological changes, it is likely that they play roles in processes including maintenance of stored sperm, egg production, immunity, and detoxification.

Several of the mating-regulated metabolism-related genes are known or predicted to promote sperm storage, egg production, and egg hatchability. For example, one such gene, *glucose dehydrogenase* (*GDH*) involved in the glucose catabolism and therefore the homeostasis of carbohydrates, is regulated by mating in female *D. melanogaster*, *Ae. aegypti*, and *Bemisia tabaci* (Hemiptera: Aleyrodidae) [29,31,39]. In *D. melanogaster*, *GDH* is expressed in the spermathecae and knockout in females leads to fewer stored sperm after mating with a previously-mated male, more asymmetrical sperm storage across spermathecae, and an elongated period of offspring production [40]. In *Ae. aegypti*, *GDH* is expressed in the spermathecal duct and the spermathecae. *GDH* knockdown in *Ae aegypti* females decreases egg hatchability, possibly through impacts on stored sperm [41]. Lida and Cavener [40] proposed that GDH may impact sperm through changing the extracellular environment of the female reproductive tract. Interestingly, in *D. melanogaster*, GDH protein is also transferred from males to females during mating suggesting that both sexes may contribute to the regulation of the female reproductive tract environment. GDH may also impact egg production through its impact on fat metabolism [42]. Hatchability is also impacted by a different mating-regulated oxidoreductase in *An. gambiae*. In this species, *heme peroxidase HPX15* expression is upregulated post-mating in glandular cells of the spermathecae and associates with stored sperm. Knockdown of *HPX15* decreases hatchability of eggs after the first gonotrophic cycle [43]. Together, these results suggest that cues received during the mating process regulate the expression of genes encoding proteins with oxidoreductase activity within the female reproductive tract and that regulation of these genes impacts the storage and maintenance of sperm in the female sperm storage organs.

In addition to oxidoreductases, other mating-regulated metabolism-related genes impact sperm storage and egg hatchability. For example, transferases are regulated by mating in several species of insects [31,34,41,44]. In *Ae. aegypti*, *N-acetylgalactosaminyl transferase 6* (*GALNT6*) is highly expressed in the spermathecal gland of unmated females but is sharply downregulated after mating. Its knockdown reduces the probability of oviposition and egg hatchability. Pascini et al. [41] proposed that *GALNT6* expression may affect the development of the spermathecae, since it is part of the chitin biosynthesis pathway [45]. Interestingly, in *D. melanogaster* a gene encoding a protein with predicted N-acetylgalactosaminyl transferase activity has highly enriched expression in the male reproductive accessory glands [46]. Therefore, as with GDH, it may be that transferases are used by both sexes to regulate processes within the reproductive tract.

The mating regulation of metabolism-related genes may also play a role in shifting female physiological processes towards egg development. For example, in several species, mating regulates the expression of dehydrogenases in metabolic pathways. As discussed above, glucose dehydrogenases are regulated by mating in *Ae. aegypti* (downregulated), *D. melanogaster* (upregulated) and the sweet potato white fly *B. tabaci* (upregulated; [29,39,41] and *glyceraldehyde 3 phosphate dehydrogenase* is upregulated by mating in *Anastrepha ludens* (Diptera: Tephritdae) [24]. The lipid biosynthesis pathway is significantly upregulated by mating in *A. ludens* and genes involved in fatty acid synthesis are upregulated by mating in both *A. ludens* and *Anastatus disparis* [24,47]. Fatty acid synthesis contributes to egg development, embryogenesis, fecundity, and digestion in some insects [48,49]. For example, in *Ae. aegypti*, knockdown of *fatty acid synthase* results in reduced fecundity and dramatically delayed blood digestion [48]. These results suggest that mating influences gene expression in such a way to affect female fecundity.

In addition to influencing reproductive processes, mating-induced regulation of metabolism-related genes may affect female ability to mount an immune response and to activate or inactivate molecules in the ejaculate. Female immune response is impacted by mating in many insect species [50,51,52]. GDH can be converted into glucose oxidase that produces hydrogen peroxide and D-gluconic acid, that are antimicrobials [53]. Insect cytochrome P450s (CYPs) are involved in the detoxification of xenobiotics, chemicals that enter their body from external sources, including insecticides and plant allelochemicals [54]. It is plausible that mating-regulation of CYPs could contribute to detoxification of seminal fluid molecules that impact female survival [23,55,56]. Further, the many proteolysis-related mating-regulated genes are likely to play a role in other processes including: immune response; the activation, inactivation, and/or degradation of seminal fluid molecules; and protection of sperm from proteolytic degradation [24,29,31,39,57]. Together, the regulation of many different genes involved in immunity and detoxification are likely to be involved in the female response to the introduction of foreign substances during the mating process.

**B.**  **Immune genes**

The importance of the regulation of the immune response in mated females is evident not only by the aforementioned mating-regulated immune function and metabolism-related genes, but also by the regulation of canonical immune genes including those encoding: heat shock proteins, thio-ester proteins, antimicrobial peptides, and ones in the JNK, Toll, IMD, Jak Stat, and RNA interference pathways [25,29,34,39,58,59,60,61]. Both across and within insect species, the direction of mating-regulation of these genes varies highlighting the complexity in understanding their roles. For example, genes encoding the antimicrobial peptide (AMP), defensin, is upregulated after mating in *Atta colombica*, *Ae. aegypti*, *B. dorsalis*, *Ceratitis capitata* (Diptera: Tephritidae), *D. melanogaster*, *Lasius niger* (Hymenoptera: Formicidae) [23,25,29,58,62,63] whereas it is downregulated after mating in *A. mellifera* [34]. Within *D. melanogaster*, *defensin* (as well as other AMP genes) expression is lower in mated females relative to unmated females at 12 h post bacterial infection but higher at 24 h post-infection [63]. Further, in *D. melanogaster*, mating-induced changes in AMP (as well as Toll and IMD) gene expression are regulated at least in part by receipt of the SFP, sex peptide [64]. These gene expression differences appear to have functional consequences in that mated *D. melanogaster* females are less able to defend against bacterial infections than unmated females [51], although more research is necessary to understand how the time-dependent changes in AMP gene expression impacts female immune response [64].

Heat shock proteins (Hsps) also impact the susceptibility of insects to pathogens and are regulated by mating in several insect species including *D. melanogaster*, *B. tabaci*, and *A. mellifera*, *An. gambiae* [23,31,33,39]. For instance, in this latter species *Hsp70b* is upregulated in the head of mated females whereas *hsp68* is downregulated in the whole body of *B. tabaci* [39,61]. Interactions between Hsps, pathogens, and immune pathways have been demonstrated in several insect species. For instance, in *Drosophila*, Hsps are involved in the activation of the nuclear factor kappa B (NF-kB) pathways (Toll and Imd) [65]. Further, the silencing of *HCS-70-4* (a member of Hsp70 family) increases the susceptibility of *D. melanogaster* to bacterial infection [65]. In *Apis mellifera*, the heat shock response repressed three AMPs (*defensin*, *abaecin* and *Hymenoptaecin*), at 45 °C as compared to 35 °C [66]. Further, the knock down of heat shock cognate 3 reduces the intensity of *Plasmodium falciparum* in *An. gambiae* [67]. Thus, mating regulation of Hsps have the potential to influence not only infection status of female insects but also their likelihood of pathogen transmission.

The potential impact of mating-regulated gene expression on pathogen transmission is also evident in the regulation of genes encoding thioester-containing proteins (TEPs). *TEP1*, for example, is upregulated in mated *An. gambiae* at 3 and 24 hpm, but not at 96 hpm [44,61]. TEP1 is secreted by mosquito hemocytes into the hemolymph and mediates killing of *P. berghei* ookinetes by binding to their surface [68]. RNAi mediated silencing of *TEP1* increases the number of oocysts in a susceptible strain of *An. gambiae* and abolishes *Plasmodium* ookinete melanization in a refractory strain, which becomes susceptible in the *TEP1*-silenced background [68]. Another gene associated with pathogen melanization, *prophenoloxidase* (PPO) and *CLIP proteases*, are also regulated by mating in several species [24,50,61,69,70,71]. Further, phenoloxidase activity increases after mating in the female of the ground cricket, *Allonemobius socius* (Orthoptera: Gryllidae) [72]. Together with the findings of physiological studies demonstrating the impact of mating on immune response, these findings on mating-regulation of immune genes support the hypothesis that mating stimulates major changes to female immunity with functional consequences for both their own health and their ability to transmit pathogens.

**C.**  **Chemosensory genes**

Chemosensation allows the transduction of environmental stimuli into signals capable of being understood by the organism. In insects, chemosensation controls crucial behaviors for survival and reproduction such as searching for food, avoiding hazards, discovering of oviposition sites, and attracting and responding to potential mates [73]. Further, chemosensory related processes change upon mating in females of some species (e.g., [74,75,76]. Chemosensory related genes are regulated by mating in females of several insect species. The main families of genes regulated by mating are those encoding odorant binding proteins (OBPs), ionotropic receptors (IRs), and gustatory receptors (GRs) [30,31,44,77,78].

Odorant binding proteins (OBPs) contribute to the transfer of odorants and pheromones to their receptors. The information received from the binding of these molecules may help insects in choosing between different stimuli such as food sources, oviposition sites, and mates [44,79]. OBPs are regulated in different directions by mating in different insect species [30,31,34,44,47]. For instance, after mating, three OBPs were downregulated in *Anastatus disparis* whereas the *OBP25* was upregulated in *An. gambiae* and *Ae. aegypti* [30,44,47]. In *Ae. aegypti*, *OBP22* was downregulated after mating, showing variation in the direction of mating regulation of different OBPs within a single species. Interestingly, knockdown of *OBP22* in *Ae. aegypti* reduces the propensity for blood meal probing [80]. This result suggests that mating-regulation of *OBP22* could be the mechanism underlying the decrease in blood feeding by female *Ae. aegypti* after mating [81,82]. More generally, investigating both the mechanisms and functional impacts of mating regulation of other OBPs could provide valuable insights for managing insect feeding and reproductive behavior.

Similarly to OBPs, ionotropic receptors (IRs) are also associated with the feeding behavior of insects. IRs that are olfactory receptors are mainly expressed in olfactory sensory neurons and allow insects to detect the volatile chemicals present in their environment [83]. For instance, in *D. melanogaster*, an IR regulates salt-induced feeding suppression [84]. Moreover, IRs are important for the detection of acids in *D. melanogaster* and their presence in sour gustatory receptor neurons (GRNs) are crucial for oviposition preference on acid containing sugar-agar as compared to sugar-agar. Indeed, in this fly, the knockout of *IR76b* and *IR25a* suppresses the responses to carboxylic and mineral acids and GRNs mediate the choice of flies to lay eggs on foods composed of acids [85]. Furthermore, some IRs are both up- and downregulated after mating in *Ae. aegypti* and *Dendrolimus punctatus* (Lepidoptera: Lasiocampidae) depending on the time point and tissue type [78]. For instance, in *Ae. aegypti, IR8a* is upregulated by mating in the head and thorax [30]. In these latter tissues, other IRs were also up- or downregulated depending on the gene but IRs in the lower reproductive tract (including all reproductive tissues except ovaries) were not regulated by mating [29,30,41]. These results are in agreement with those obtained in *D. punctatus* where different individual IRs are also upregulated by mating in antennae [78]. Thus, mating may regulate IR gene expression in such a way that modifies female response to volatile cues related to feeding and reproduction.

Like OBPs and IRs, gustatory receptors (GRs) are involved in chemosensation and are regulated in both directions by mating. These G-protein coupled receptors are transmembrane molecules expressed in gustatory receptor neurons of insects where they respond to various attractants such as sweat, lactic acid, octenol, and carbon dioxide (CO_2_) [86]. In *D. melanogaster* and *Ae. aegypti*, the CO_2_ receptors are composed of GRs. Silencing of these genes in *Ae. aegypti* lead to the loss of CO_2_ sensitivity, which could change female attraction to blood meal sources [87]. Furthermore, the expression of GRs leads to a significant response to the insecticide canavanine in low salt sensing GR-expressing neurons and the disruption of GRs in *D. melanogaster* produces an inability to avoid this aversive compound [88,89]. These latter examples show the implication of GRs in the detection and susceptibility of several compounds in insects. In this way, by regulating expression of GR-encoding genes, mating may regulate female responsiveness to different stimuli. Mating regulates the expression of genes encoding GRs (in both directions) in the antennae of *D. punctatus*. For instance, *gustatory receptor for sugar taste 64a* is downregulated while *gustatory receptor 4* is upregulated [78], but the specific functions of these GRs is not yet known. Therefore, further studies should be conducted to decipher the role and the mechanism of action of GRs that are differentially expressed during mating in insects. Furthermore, mating-regulated genes encoding chemosensation related proteins are excellent candidates for investigating how mating changes female response and receptivity to further courtship and mating attempts by males.

## 3. Studies Examining the Functional Consequences of Mating-Regulated Gene Expression Changes

After determining which genes are regulated by mating, a next step is to investigate the effects of this mating-induced gene regulation on female phenotypes. One excellent example of such an investigation is the series of studies by Catteruccia and colleagues on *mating-induced stimulator of oogenesis* (*MISO*, AGAP002620) in *An. gambiae* [57,90]. In this species, studies of mating-induced gene expression changes in females, combined with subsequent biochemical and functional studies, led to the identification of *MISO* that is regulated by the receipt of seminal fluid 20-hydroxyecdysone (20E) by females [57,90,91]. Knockdown of *MISO* leads to a reduction of the mating-induced egg production by delaying the development of follicles and by impeding the release of 20E from the atrium and subsequent expression of 20E-regulated genes [90,91]. Furthermore, *MISO* expression protects females from a *Plasmodium* infection induced reduction in egg production [92]. These careful studies, together with others from the Catteruccia lab, have demonstrated how a detailed understanding of the mechanisms underlying mating-induced changes can provide important insights into the molecular social interactions between males and females and between a pathogen and host [93].

*Drosophila melanogaster* provides another example of how mating-regulated gene expression studies can result in a more thorough understanding of post-mating phenotypic changes. In this species, the identification of musculature-related genes regulated by mating [59] led to novel discoveries of the impact of mating on morphological and physiological changes in the female reproductive tract [94]. These post-mating changes include promotion of innervation and muscle differentiation in the oviduct, opening of the oviduct lumen, and relaxation of the oviduct musculature [20,94], which presumably function to facilitate release of eggs from the ovary to the uterus. These changes to the oviduct are likely mediated, at least in part, by the mating-induced increase release of neuromodulators, including octopamine [19,95,96]. In particular, the ovulation-inducing SFP, ovulin, appears to exert its effect by increasing octopamine signaling within the female reproductive tract [20]. Further studies have demonstrated other major post-mating structural changes to the female reproductive tract [97,98,99]. Thus, in the case of *D. melanogaster*, studies of mating- (and SFP-) regulated gene expression complemented extensive elegant biochemical, physiological, and morphological studies to further our identification of female post-mating phenotypic changes and their underlying mechanisms.

## 4. Discussion and Future Directions

**Figure 1:**
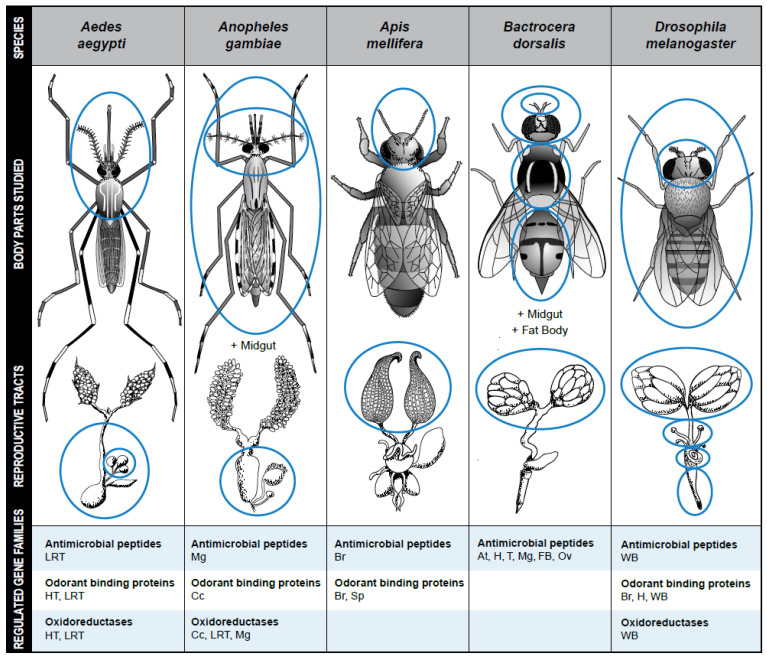
Examples of common gene families regulated by mating in five well-studied insect species. Body parts and reproductive tissues in which mating-regulated gene expression have been studied are encircled. At = Antennae; Cc = Carcass; Br = Brain; FB = Fat body; H = Head; HT = Head and Thorax; LRT = Lower Reproductive tract; Mg = Midgut; Ov = Ovary; Sp = Spermathecae; T = Thorax; WB = whole body. Design, graphics, and drawings by Jodi Robison and Leslie Weekley.

The goal of this review was to summarize the current state of knowledge about common gene families regulated by mating in female insects. The most common mating-regulated gene families in females are predicted to be involved in three main functions: metabolism, immunity, and chemosensation (Figure 1). Although different protocols and techniques are used during gene expression studies, for each function, some gene or gene families that are differentially expressed are shared by at least two species. For example, *OBP25*, which encodes a protein involved in chemosensation, is significantly upregulated in the mated females of *An. gambiae* and *Ae. aegypti* [30,44]. Contrary to *OBP25* that has the same pattern across insect species, the immune gene *defensin* has a different pattern of regulation in different species but it is regulated by mating in females of at least six species including *Atta colombica* (Hymenoptera: Formicidae), *Ae. aegypti*, *C. capitata*, *D. melanogaster*, *L. niger* and *A. mellifera* [23,29,34,58,62,63]. Like *defensin*, *GDH* that plays a role during insect metabolism has a different regulation pattern after mating in *D. melanogaster*, *Ae. aegypti*, and *B. tabaci* [29,31,39,41]. These genes that are differentially expressed, especially those that are upregulated by mating across different insects are good candidates for insect management and a standardization of protocols may confirm their roles in more insects.

Understanding how mating and specific cues received during mating impact female phenotypes is critically important both for a basic understanding of insect physiology, molecular biology, and behavior and for solving applied problems including controlling reproduction and lifespan for pest management and conservation breeding. One way to better understand the phenotypes that are impacted by mating and the mechanisms underlying these impacts is to analyze which genes change expression after mating. In this field of post-mating changes in female insects, gene expression studies are increasingly available, but it is difficult to compare findings between studies because of lack of standardization between them. Subsequent studies should work to establish norms and standards for gene expression studies and this field of study will progress more efficiently by collaborating across multiple species.

Although there now are a number of studies of the effects of mating on gene expression in female insects, the results of these studies are difficult to compare and draw broad conclusions from due to variation in methodologies between studies (Table 1). For example, the studies we reviewed varied in timepoints post mating; whether or not matings were observed to establish precise timepoints; tissues analyzed; and methods for RNA extraction and differential gene expression analysis. With the goal of developing our understanding of general and species-specific effects of mating on gene expression, we suggest the following considerations prior to new studies:

1. Time points after mating: Most studies that we reviewed either used set time points after mating of 0 h, 6 h, and 24 h, or placed females with males but did not actually record exact latencies between mating and mRNA extraction. We recommend using set time points after observed matings rather than putting females with males unobserved as the patterns of gene expression change dramatically with time since mating even over the course of a few hours. Furthermore, it would be interesting and important to explore in more species whether there are additional changes in gene expression patterns that occur at later time points, as some post-mating phenotypic changes occur and/or persist over the course of several days to months (e.g., for species in which females reject male mating attempts for their entire lives) or possibly years (e.g., in honey bee, *A. mellifera*, queens) [100].
2. Number of matings: All studies that we reviewed either compared unmated versus singly mated females or compared unmated females with females that had an unknown number matings because they were left unobserved with males. Females of many insect species mate multiple times. The phenotypic effects of single versus multiple matings for females have not been as well explored as the effects of single versus no mating. It would be a useful addition to the field of insect reproduction to understand how variations in female reproductive history (e.g., number and frequency of matings; operational sex ratio; density) impact both female post-mating phenotypes and gene expression.
3. Tissues for analysis: Just as gene expression varies between tissue types, expression changes in response to mating are likely to be tissue-specific. Important patterns of mating-induced gene expression changes might be obscured when analysis is at the level of the whole body. Resources-permitting, the field would benefit from having within species studies documenting patterns of mating-induced gene expression across different tissue types expected to be relevant to post-mating phenotypic changes (e.g., nervous system tissues; fat bodies; ovaries; sperm storage organs). Further, comparisons of mating-induced gene expression patterns within the same tissues across species would provide insights into the consistent and divergent genes that are regulated by mating.
4. RNA extraction methods: The methods used for RNA extraction can potentially impact the discovery rate of mating-induced gene expression changes. For example, a comparison of three different RNA extraction methods in the yeast, *Saccharomycies cerevisiae*, yielded significantly different transcript abundances between phenol-extracted samples relative to samples extracted using two commercially available kits. These differential transcript abundances could potentially impact the detection of differentially expressed genes when comparing between treatments [101].
5. Sequencing and analysis methods: In the case of RNAseq, methodology for conducting, sequencing and analyzing gene expression studies is improving rapidly. Therefore, it is not possible to recommend a single methodology that will remain the best approach over time. However, it is important to note that the analysis method can affect the results [102] and therefore care should be taken when interpreting comparisons across studies that used different sequencing and analysis methods. Appropriate and/or best practices should be established for differential expression studies in each study species and may vary depending on that species’ genome or transcriptome features [103].
6. Functional analyses: Studies of mating-induced gene expression pattern changes provide a good starting point both for generating hypotheses for mechanisms underlying known post-mating phenotypic changes and for discovering novel post-mating phenotypic changes. However, future studies should also investigate the functional consequences of mating-induced changes in gene expression on female phenotypes, similar to the studies described above in *An. gambiae* and *D. melanogaster*. Such functional studies will provide insights into the consequences of intersexual molecular interactions and could be important for understanding both basic biological processes and applied insect management program that depend on reproductive processes such as the sterile insect technique and release of insects carrying a dominant lethal.

**Table 1:** Comparison of Approaches to Analyze Gene Expression Regulation Between Microarrays, qPCR and RNAseq. Nb for number. Cq stands for quantification cycle.

Technologies/Parameters	Microarrays	qPCR	RNAseq
**Description**	Comparison of expression levels of predefined genes	Transcript quantification/expression of predefined genes in real time	RNA sequencing and gene quantification/expression of several genes
**Principle**	Hybridization	PCR and Cq	Deep sequencing
**Sensitivity**	Intermediate	Highest	Lowest
**Specificity**	Intermediate	Highest	Lowest
**Nb of genes studied**	Limited	Limited	Unlimited
**Background signal**	Low	Low	Very low
**Transcription abundance detection**	Intermediate	Highest	High
**Time**	Long	Short	Long
**Price**	Expensive	Less expensive	Expensive
**Expertise Needed**	High	Intermediate	Highest
**Examples**	*An. gambiae* *Ceratitis capitata* *D. melanogaster*	*An. gambiae* *An. coluzzii* *Ae. aegypti* *Anastatus disparis* *Apis mellifera* *Bactrocera dorsalis* *Ceratitis capitata* *Dendrolimus punctatus*	*An. gambiae* *An. coluzzii* *Ae. aegypti* *Anastatus disparis* *Apis mellifera* *Bactrocera dorsalis* *Bactrocera tryoni* [104] *Dendrolimus punctatus* *D. melanogaster* *Anastrepha ludens*

**Collaborations to progress our understanding of mating-induced gene expression changes.**

The field of post-mating gene expression changes would benefit greatly from a large-scale collaboration between researchers studying different insect species and between scientists working in different sub-disciplines of biology. Such a collaboration could progress the field forward by benefiting from specific knowledge of the reproductive biology, behavior, physiology, and natural history of different insect species coupled together with specialized expertise in fields such as insect cell biology, immunology, biochemistry, evolution, and bioinformatics. This approach could be used to understand important questions such as those explained below.

- Across different species and different mating/social systems, are the similar patterns of post-mating female responses (e.g., egg development, mating inhibition) induced by regulation of the same genes or pathways? If so, that would suggest that these phenotypic changes may be conserved and homologous. In contrast, if the genes or pathways are different, it would suggest that these similar post-mating phenotypes arose through multiple independent evolutionary events (i.e., convergent evolution). Such a finding would lay the groundwork for further research on what common selective forces result in the evolution of particular post-mating females responses and answer questions such as whether mating induced gene expression changes correspond to particular life or natural history characteristics (e.g., monandrous vs. polyandrous; degree of sociality; diet; how individuals find mates; lifespan).
- What are the mating-related stimuli (e.g., mechanical, chemical, molecular) that induce gene expression changes in females and are these similar or divergent across species? Intriguingly, some of the most thoroughly studied stimuli of female post-mating changes (e.g., *D. melanogaster* sex peptide and ovulin) induce only subtle effects on gene expression patterns [64,105] and there is evidence that sex peptide induces its impacts through regulating expression of microRNAs [106]. By identifying these stimuli, we can begin to explore the mechanisms by which an externally-derived stimulus from a male (e.g., sperm, seminal fluid, vibrations, or odors) can affect the gene expression changes of a female in such a way that induces profound behavioral and/or physiological changes as well as the behavioral and molecular interactions between females and males that result in these changes [93,107]. Through such studies, we then may be able to devise approaches for manipulating female gene expression changes either directly or indirectly through artificial selection or transgenesis of male behavior or physiology.

## Data Availability

Not applicable.
